# Imatinib after induction for treatment of children and adolescents with Philadelphia-chromosome-positive acute lymphoblastic leukaemia (EsPhALL): a randomised, open-label, intergroup study

**DOI:** 10.1016/S1470-2045(12)70377-7

**Published:** 2012-09

**Authors:** Andrea Biondi, Martin Schrappe, Paola De Lorenzo, Anders Castor, Giovanna Lucchini, Virginie Gandemer, Rob Pieters, Jan Stary, Gabriele Escherich, Myriam Campbell, Chi-Kong Li, Ajay Vora, Maurizio Aricò, Silja Röttgers, Vaskar Saha, Maria Grazia Valsecchi

**Affiliations:** a Department of Paediatrics, University of Milano-Bicocca, Monza, Italy; b Department of Clinical and Preventive Medicine and EsPhALL Trial Data Centre, University of Milano-Bicocca, Monza, Italy; c Department of Paediatrics, University Medical Centre and Christian-Albrechts-University, Kiel, Germany; d Paediatric Haematology Oncology, Lund University Hospital, Lund, Sweden; e Department of Paediatric Haematology Oncology, CHU Hôpital Sud, Rennes, France; f Erasmus MC–Sophia Childrens Hospital, University Medical Centre Rotterdam, Rotterdam, Netherlands; g Department of Paediatric Haematology and Oncology, University Hospital Motol, Prague, Czech Republic; h University Medical Centre Hamburg-Eppendorf, Clinic of Paediatric Haematology and Oncology, Hamburg, Germany; i Division of Paediatric Haematology-Oncology, Hospital Roberto del Río, University of Chile, Santiago, Chile; j Department of Paediatrics, Prince of Wales Hospital, Shatin, China; k Sheffield Children's Hospital, Sheffield, UK; l Paediatric Haematology Oncology, Azienda Ospedaliero-Universitaria Meyer, Florence, Italy; m Oncogenetic Laboratory, Paediatric Haematology and Oncology, Justus-Liebig University, Giessen, Germany; n Paediatric and Adolescent Oncology, Central Manchester University Hospital Foundations Trust, Manchester Academic Health Sciences Centre, School of Cancer and Enabling Sciences, University of Manchester, UK

## Abstract

**Background:**

Trials of imatinib have provided evidence of activity in adults with Philadelphia-chromosome-positive acute lymphoblastic leukaemia (ALL), but the drug's role when given with multidrug chemotherapy to children is unknown. This study assesses the safety and efficacy of oral imatinib in association with a Berlin–Frankfurt–Munster intensive chemotherapy regimen and allogeneic stem-cell transplantation for paediatric patients with Philadelphia-chromosome-positive ALL.

**Methods:**

Patients aged 1–18 years recruited to national trials of front-line treatment for ALL were eligible if they had t(9;22)(q34;q11). Patients with abnormal renal or hepatic function, or an active systemic infection, were ineligible. Patients were enrolled by ten study groups between 2004 and 2009, and were classified as good risk or poor risk according to early response to induction treatment. Good-risk patients were randomly assigned by a web-based system with permuted blocks (size four) to receive post-induction imatinib with chemotherapy or chemotherapy only in a 1:1 ratio, while all poor-risk patients received post-induction imatinib with chemotherapy. Patients were stratified by study group. The chemotherapy regimen was modelled on a Berlin–Frankfurt–Munster high-risk backbone; all received four post-induction blocks of chemotherapy after which they became eligible for stem-cell transplantation. The primary endpoints were disease-free survival at 4 years in the good-risk group and event-free survival at 4 years in the poor-risk group, analysed by intention to treat and a secondary analysis of patients as treated. The trial is registered with EudraCT (2004-001647-30) and ClinicalTrials.gov, number NCT00287105.

**Findings:**

Between Jan 1, 2004, and Dec 31, 2009, we screened 229 patients and enrolled 178: 108 were good risk and 70 poor risk. 46 good-risk patients were assigned to receive imatinib and 44 to receive no imatinib. Median follow-up was 3·1 years (IQR 2·0–4·6). 4-year disease-free survival was 72·9% (95% CI 56·1–84·1) in the good-risk, imatinib group versus 61·7% (45·0–74·7) in the good-risk, no imatinib group (p=0·24). The hazard ratio (HR) for failure, adjusted for minimal residual disease, was 0·63 (0·28–1·41; p=0·26). The as-treated analysis showed 4-year disease-free survival was 75·2% (61·0–84·9) for good-risk patients receiving imatinib and 55·9% (36·1–71·7) for those who did not receive imatinib (p=0·06). 4-year event-free survival for poor-risk patients was 53·5% (40·4–65·0). Serious adverse events were much the same in the good-risk groups, with infections caused by myelosuppression the most common. 16 patients in the good-risk imatinib group versus ten in the good-risk, no imatinib group (p=0·64), and 24 in the poor-risk group, had a serious adverse event.

**Interpretation:**

Our results suggests that imatinib in conjunction with intensive chemotherapy is well tolerated and might be beneficial for treatment of children with Philadelphia-chromosome-positive ALL.

**Funding:**

Projet Hospitalier de Recherche Clinique-Cancer (France), Fondazione Tettamanti-De Marchi and Associazione Italiana per la Ricerca sul Cancro (Italy), Novartis Germany, Cancer Research UK, Leukaemia Lymphoma Research, and Central Manchester University Hospitals Foundation Trust.

## Introduction

Although survival of children with acute lymphoblastic leukaemia is almost 85%, outcome for the 3–5% of patients with Philadelphia-chromosome-positive acute lymphoblastic leukaemia (ALL) is poor.^1^, ^2^ An international study^3^ reported results for 610 children treated with intensive chemotherapy without tyrosine-kinase inhibitors. 7-year event-free survival was 32% and overall survival was 45%. Matched donor allogeneic stem-cell transplantation was beneficial. Many groups treating adults with Philadelphia-chromosome-positive acute lymphoblastic leukaemia have noted the safety and effectiveness of imatinib^4^, ^5^ when given with chemotherapy.^6^, ^7^, ^8^, ^9^, ^10^, ^11^, ^12^, ^13^, ^14^, ^15^, ^16^, ^17^, ^18^, ^19^, ^20^ In an observational study,^21^ the Children's Oncology Group (COG) assessed increased exposure to imatinib combined with chemotherapy in five cohorts. 44 children who received continuous imatinib from consolidation to the end of treatment, had a 3-year event-free survival of 80%, with acceptable toxic effects. In this group, which excluded patients with induction failure, outcome with matched donor allogeneic stem-cell transplantation was not better than chemotherapy plus imatinib. In a small number of patients treated with a high-risk chemotherapy protocol (SHOP-2005) plus imatinib in Spain, outcome was good compared with historical controls.^22^ Minimal residual disease at the end of induction is an independent predictor of outcome in childhood acute lymphoblastic leukaemia.^23^ However, in the COG study, minimal residual disease assessed by multiparameter flow cytometry at the end of induction treatment, before administration of imatinib, was not prognostic for survival.

We report results of the European intergroup study of post-induction treatment of Philadelphia-chromosome-positive ALL (EsPhALL), which is contemporary to the COG study. The aim was to test the safety and long-term efficacy of post-induction imatinib plus chemotherapy compared with the standard Berlin–Frankfurt–Munster backbone intensive treatment, using a risk-stratified approach for treatment of patients on the basis of early response to therapy.^24^

## Methods

### Patients

To provide adequate statistical power, the study was started as a large collaborative trial. Patients were enrolled into this open-label, randomised trial by ten national study groups, mainly in Europe (AIEOP, BFM-G/CH, COALL, FRALLE, NOPHO, MRC, DCOG, CPH, PINDA, and HKPHOSG), which obtained ethics approval from their own institutions.

Patients aged 1–18 years recruited to national trials of front-line treatment for ALL were eligible if t(9;22)(q34;q11) was present according to cytogenetics tested at the participating institution and the presence of a *BCR-ABL* fusion transcript identified by real-time PCR—detecting the transcripts for both the p190 and p210 isoforms—or fluorescence in-situ hybridisation (FISH). Information about additional cytogenetic abnormalities was not recorded, because it was beyond the scope of the study. Patients with abnormal renal or hepatic function (grade 2–3 toxic effects according to the National Cancer Institute Common Toxicity Criteria [NCI CTC]) or active systemic infection at the end of induction treatment were ineligible. Written, informed consent was obtained from parents or legal guardians.

After induction treatment, patients were classified as good risk or poor risk. Good-risk patients were those who had both early response and complete remission at the end of induction. Early response was defined as blast cell count of less than 1000 cells per μL in peripheral blood after 7 days of treatment with prednisone and a single intrathecal dose of methotrexate, or 25% or less bone marrow blast cells at day 15, or less than 5% bone marrow blast cells at day 21, depending on national induction protocols. Complete remission was defined as less than 5% blast cells in bone marrow aspirate (with absolute neutrophil count ≥1500 cells per μL and ≥100 000 platelets per μL) and absence of leukaemia in other organs. Poor-risk patients were those who did not have early response or complete remission at the end of induction, and received chemotherapy and imatinib.

### Randomisation and masking

Patients classified as good-risk patients were randomly assigned to receive imatinib with chemotherapy or chemotherapy only. We did the randomisation with a centralised web-based randomisation system^25^ with permuted blocks (size four) and a 1:1 allocation ratio. The allocation was stratified by study group to account for the different methods of assessment of early response. When a study centre identified a potential patient, eligibility was confirmed by the group data centre. The study was open label.

### Procedures

**Figure 1:**
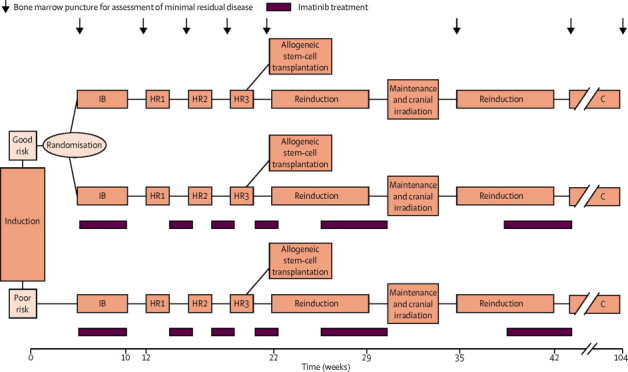
Study design For details of the chemotherapy regimens see appendix. C=continuation therapy.

Chemotherapy was modelled on a Berlin–Frankfurt–Munster high-risk backbone,^24^ with all patients receiving four post-induction blocks of treatment—protocol IB, HR1, HR2, and HR3 (appendix)—after which they became eligible for allogeneic stem-cell transplantation in complete remission (figure 1, appendix).^1^ Stem-cell transplantation was recommended for all poor-risk patients and for good-risk patients with any genotype-matched donor. Transplantation from both matched related and unrelated donors was allowed in good-risk patients because the two techniques seem to have much the same outcome in Philadelphia-chromosome-positive ALL as a result of improved HLA matching and supportive care.^3^, ^26^ Patients who did not have a transplant had cranial irradiation and two re-induction blocks before continuation of treatment. Preliminary data from a phase 1 study^27^ show that daily oral imatinib is well tolerated in children at doses of 260–570 mg/m^2^ per day, therefore we used an intermediate dose of 300 mg/m^2^ per day for our study. Imatinib was given for 126 days, starting from the end of induction, concomitantly with chemotherapy during protocol IB and in an alternated regimen with the other protocols (figure 1), as done in contemporary studies of adults (appendix).^17^, ^20^

Resistance to protocol was defined as 5% blast cells or more in bone marrow aspirate at the end of the third consolidation block of treatment for those without previous complete remission. Late response was defined as less than 5% blast cells in bone marrow aspirate at the end of either protocol IB or one of three consolidation blocks, for patients who did not have complete remission at end of induction. Bone marrow relapse was defined as 25% blast cells or more in bone marrow, after remission.

Minimal residual disease was analysed in central laboratories (one for each study group) that agreed on standard procedures. It was assessed in bone marrow and peripheral blood cells collected at eight points (figure 1), by quantitative real-time PCR amplification of immunoglobulin, T-cell receptor gene,^28^ and *BCR-ABL* fusion gene, according to guidelines.^29^ Few patients had data for the *BCR-ABL* fusion gene and so they were not included in the analysis, mainly because of scarcity of RNA for analysis. We report only immunoglobulin and T-cell receptor data measured after induction, according to the cutpoint usually adopted to distinguish high-risk patients in AIEOP-BFM ALL protocols.^30^, ^31^

Serious adverse events were reported to the national contact person within 24 h and monitored at follow-up by the coordination unit (AB, MS, MGV, and PDL). Safety was assessed by clinical observation and weekly haematological and biochemical monitoring.

The primary endpoint in the good-risk group was disease-free survival at 4 years because the group only includes patients in first complete remission. For poor-risk patients the primary endpoint was event-free survival at 4 years. The secondary endpoint was overall survival at 4 years. The main analyses were done by intention to treat; a secondary analysis was done of patients as treated.

Disease-free survival was defined as time from randomisation (or date of complete remission for poor-risk patients) until relapse at any site, death during complete remission, or development of a second malignant neoplasm. Event-free survival was defined as time from start of treatment (ie, protocol IB) to first failure, defined as resistance, relapse, death from any cause, or second malignant neoplasm. Overall survival was defined by the time from start of treatment to death from any cause.

### Statistical analysis

The international trial data centre checked the quality of data and produced blinded yearly reports for the trial's steering committee and interim analyses for the data monitoring committee.

The study aimed to recruit 140 good-risk patients, to provide 80% power to detect a 24% difference in 4-year disease-free survival, with an expected baseline disease-free survival of 40% (two-sided test, 5% type 1 error and a minimum of 2 years of follow-up), according to Lachin and Faulkes.^32^ During 2009, the steering committee reviewed preliminary study results and external evidence for the benefit of imatinib^21^ and decided to prematurely stop enrolment. Final follow-up was on Dec 31, 2010.

Observation periods were censored at date of last contact when no events were reported. We produced Kaplan-Meier curves for each endpoint (with Greenwood SEs^33^) and compared groups with the log-rank test. We applied the Cox model to estimate hazard ratios (HRs; tested according to Wald). For the regressor in the model we included the variables treatment and post-induction minimal residual disease (three categories: <5×10^−4^ cells, ≥5×10^−4^ cells, and unknown) because of an unexpected imbalance of minimal residual disease in the randomised groups. Planned secondary analyses were by group at 2 years after censoring patients who had transplants at date of stem-cell transplantation, and as-treated analyses counting deviations from the assigned group and comparing patients who actually received imatinib with those who did not. We calculated the probabilities of relapse and of death in continuous complete remission with the cumulative incidence estimator, thus allowing for competing risks, and compared them with the Gray test. We used the χ^2^ test to assess the association between treatment group and severe adverse events. All tests were two sided. We did all analyses with SAS (version 9.2). This trial is registered with the EudraCT (2004-001647-30) and ClinicalTrials.gov, number NCT00287105.

### Role of the funding source

Novartis provided the study drug. The sponsors had no role in study design, data collection, data analysis, data interpretation, or writing of the report. All authors had full access to all the data in the study and had final responsibility for decision to submit for publication.

## Results

**Figure 2:**
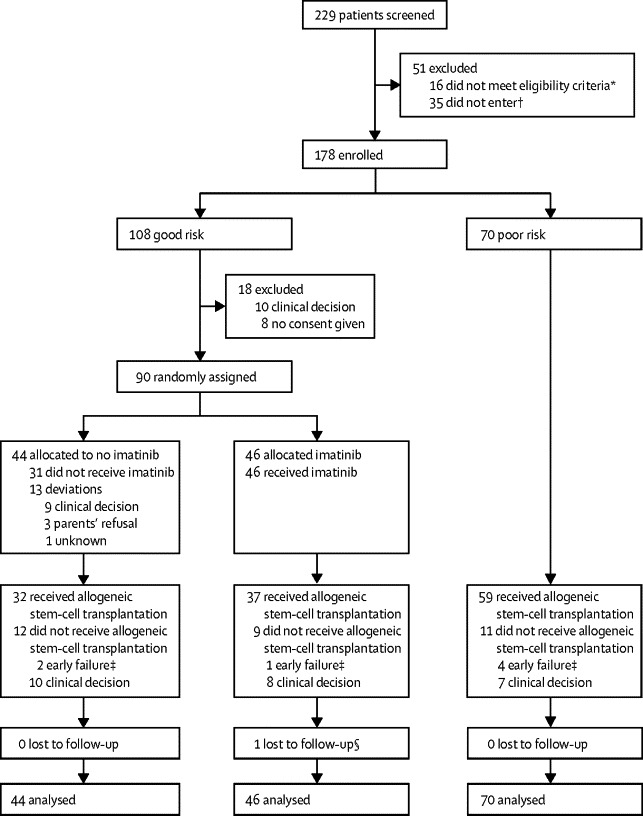
Trial profile *12 not enrolled in front-line acute lymphoblastic leukaemia protocols, two did not have relevant data, one had abnormal renal function, and one had active fungal infection. †For 24 patients trial protocol not approved by ethics committee, four because of clinical decision, three because of parents' refusal, two because of late diagnosis of t(9;22), and two died during induction. ‡Early failures were defined as relapses and deaths during continuous complete relapse within 5 months of first complete remission. §One patient lost to follow-up because of infection with *Clostridium* sp not related to imatinib (patient had liver failure, disseminated intravascular coagulation, and lung consolidation).

Recruitment started on Jan 1, 2004, and ended on Dec 31, 2009. 229 patients were screened, 213 met eligibility criteria, and 178 (age range 1·5–17·9 years) were enrolled and stratified as good risk (108; 61%) or poor risk (70; 39%; figure 2). Up to 2008 (when evidence for adults became fully available), eight of 90 good-risk patients (9%) refused to be assigned, in 2009, ten of 18 eligible good-risk patients (56%) were not assigned, resulting in 90 good-risk patients being randomly assigned. More patients were older than 10 years in the poor-risk group than in the good-risk group and fewer patients had white blood cell counts of less than 50 cells per μL at diagnosis (table 1).

**Table 1:** Baseline characteristics

		**Good-risk, imatinib group (n=46)**	**Good-risk, no imatinib group (n=44)**	**Poor-risk group (n=70)**
Female		17 (37%)	16 (36%)	26 (37%)
Male		29 (63%)	28 (64%)	44 (63%)
Age at diagnosis				
	<10 years	28 (61%)	28 (64%)	29 (41%)
	≥10 years	18 (39%)	16 (36%)	41 (59%)
White blood cell count (cells per μL)				
	Data not available	0	2	1
	<50	29 (63%)	25 (60%)	20 (29%)
	50–100	6 (13%)	6 (14%)	14 (20%)
	>100	11 (24%)	11 (26%)	35 (51%)
Immunophenotype				
	Data not available	0	0	1
	Common	21 (46%)	19 (43%)	41 (59%)
	Pre-B	20 (43%)	19 (43%)	19 (28%)
	Pro-B	1 (2%)	0 (0%)	3 (4%)
	Other B cell precursor	4 (9%)	5 (11%)	5 (7%)
	T-cell lineage	0 (0%)	1 (2%)	1 (1%)
CNS involvement				
	Not assessable	1 (2%)	2 (5%)	4 (6%)
	Yes	4 (9%)	4 (9%)	4 (6%)
	No	41 (89%)	38 (86%)	62 (89%)
t (9;22)(q34;q11)				
	Fluorescence in-situ hybridisation only	14 (30%)	18 (41%)	23 (33%)
	Real-time PCR only	10 (22%)	11 (25%)	17 (24%)
	Both	22 (48%)	15 (34%)	30 (43%)
If real-time PCR, transcript detected				
	Data not available	7	6	7
	p190	23 (92%)	18 (90%)	31 (78%)
	p210	2 (8%)	2 (10%)	9 (23%)
Early response*				
	Yes (peripheral blood)	23 (50%)	22 (50%)	1 (1%)
	No (peripheral blood)	0 (0%)	0 (0%)	39 (56%)
	Yes (bone marrow)	23 (50%)	22 (50%)	3 (4%)
	No (bone marrow)	0 (0%)	0 (0%)	27 (39%)
Minimal residual disease at end of induction†				
	Data not available	16	21	23
	<5×10^−4^	11 (37%)	15 (65%)	2 (4%)
	≥5×10^−4^	19 (63%)	8 (35%)	45 (96%)

* Early response was assessed in bone marrow in COALL, FRALLE, MRC, and NOPHO, and in peripheral blood in the other groups.

† Hong-Kong and PINDA did not contribute data.

All patients except two had progenitor-B Philadelphia-chromosome-positive acute lymphoblastic leukaemia. Of 85 patients for whom detection of p190 and p210 was possible, p210 was detected more often in poor-risk patients than in good-risk patients.

Four patients achieved early response but not complete response at the end of induction and were classified as poor risk. Minimal residual disease at the end of induction was higher in poor-risk patients than in good-risk patients.

Baseline characteristics were much the same in each good-risk group, except for post-induction minimal residual disease, with low levels (<5×10^−4^) more frequent in the good-risk, no imatinib than in the good-risk, imitinab (table 1). Median follow-up of the 178 enrolled patients was 3·1 years (2·0–4·6). For these patients 4-year event-free survival was 61·9% (95% CI 52·2–69·8) and 4-year overall survival was 72·1% (95% CI 64·5–79·7). For the 160 patients who were randomly assigned or in the poor-risk group, 4-year event-free survival was 61·0% (95% CI 53·0–69·0) and overall survival was 72·2% (95% CI 64·4–80·0).

Allogeneic stem-cell transplantation was done in first complete remission in 137 of 178 (77%) patients (78 with good risk, 59 with poor risk) at a median of 155 days from first complete remission, and after the HR3 block for 112 (82%) patients (table 2). The most common type of transplant was from HLA-matched unrelated donors in both risk groups (44 [56%] good risk, 27 [46%] poor risk).

**Table 2:** Relapses and deaths

		**Good-risk, imatinib group**		**Good-risk, no imatinib group**		**Poor-risk group**	
		Chemotherapy (n=9)*	Allogeneic stem-cell transplantation (n=37)	Chemotherapy (n=12)*	Allogeneic stem-cell transplantation (n=32)	Chemotherapy (n=11)*	Allogeneic stem-cell transplantation (n=59)
First relapse		4 (44%)	6 (16%)	5 (42%)	7 (22%)	7 (64%)	16 (27%)
Site of first relapse							
	Bone marrow	2 (22%)	4 (11%)	4 (33%)	7 (22%)	4 (36%)	13 (22%)
	Bone marrow and other	1 (11%)	2 (5%)	0 (0%)	0 (0%)	2 (18%)	2 (3%)
	Extramedullary†	1 (11%)	0 (0%)	1 (8%)	0 (0%)	1 (9%)	1 (2%)
Death in continuous complete remission		0 (0%)	2 (5%)	1 (8%)	3 (9%)	2 (18%)	6 (10%)
Cause of death							
	Graft-versus-host disease	0 (0%)	1 (3%)	0 (0%)	2 (6%)	0 (0%)	0 (0%)
	Infection	0 (0%)	1 (3%)	1 (8%)	1 (3%)	0 (0%)	4 (7%)
	Other‡	0 (0%)	0 (0%)	0 (0%)	0 (0%)	2 (18%)	2 (3%)
Alive in continuous complete remission§		0 (0%)	2 (5%)	1 (8%)	3 (9%)	2 (18%)	6 (10%)

Data are n (%). 108 of 128 randomised patients (84%) had allogeneic stem-cell transplantation after HR3, eight (6%) before HR3, and seven (5%) after additional consolidation (data were unavailable for five patients).

* Includes patients who failed early—ie, relapsed or died in complete continuous remission within 5 months of complete remission (one relapse in the good-risk, imatinib group; one relapse and one death in the good-risk, no imatinib group; and two relapses and two deaths in the poor-risk group).

† All in CNS, except for one in testis in the good-risk, imatinib group.

‡ Other causes of death were capillary leak syndrome (n=1) and cardiac failure during chemotherapy (n=1). Severe autoimmune diseases (n=1) and intracranial bleeding (n=1) occurred after transplantation.

§ No second malignant neoplasms were reported during the follow-up.

**Figure 3:**
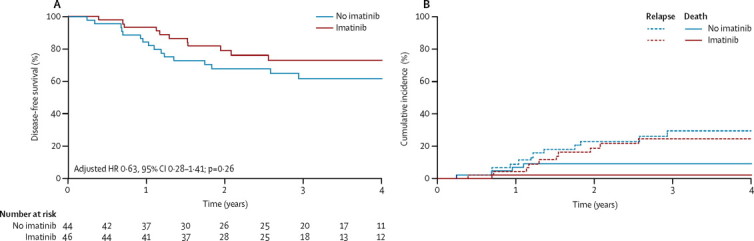
Disease-free survival and cumulative incidence of relapse and of death in continuous complete remission in good risk patients, analysed by intention to treat (A) Disease-free survival. (B) Cumulative incidence of relapse and death continuous complete remission for patients in the good-risk group. One event in a patient in the imatinib group at 6 years after randomisation is omitted (died in continuous complete remission of pulmonary graft-versus-host disease after transplantation).

At 4 years, disease-free survival was 72·9% (95% CI 56·1–84·1) in the good-risk, imatinib group and 61·7% (45·0–74·7) in the good-risk, no imatinib group (p=0·24), with an absolute difference of 11·2% (95% CI −9·2 to 31·6; figure 3A) when calculated by intention to treat. The HR for any event was 0·71 (95% CI 0·33–1·54; p=0·38) and was 0·63 (95% CI 0·28–1·41; p=0·26) when adjusted for minimal residual disease after induction. 4-year overall survival was 85·1% (95% CI 69·6–93·1) in the good-risk, imatinib group and 72·9% (53·9–85·0) in the good-risk, no imatinib group (p=0·37). The HR for death was 0·68 (95% CI 0·26–1·81; p=0·44) and 0·59 (0·21–1·65; p=0·31) when adjusted for minimal residual disease after induction.

Relapse was the most frequent event in both good-risk groups, involving the bone marrow in all except four patients and half of relapses occurred after stem-cell transplantation, which was done in 37 (80%) of 46 patients in the good-risk, imatinib group versus 32 (73%) of 44 patients in the good-risk, no imatinib group at a median time of 5·3 months (IQR 4·8–6·4) versus 5·4 months (4·7–6·1). Cumulative incidence of relapse at 4 years was 24·8% (95% CI 11·1–38·5) in the good-risk, imatinib group and 29·2% (14·9–43·5) in the good-risk, no imatinib group (p=0·66) and all relapses occurred within 3 years of entry into the study (figure 3B). After relapse, roughly half of patients died, mainly from disease progression. More deaths in continuous complete remission occurred in patients in the good-risk, no imatinib group than in the good-risk, imatinib group and none were related to imatinib (table 2).

When censored at stem-cell transplantation during first complete remission, 2-year disease-free survival was 81·2% (95% CI 30·7–96·4) in the good-risk, imatinib group versus 65·4% (30·4–86·0) in the good-risk, no imatinib group (p=0·97). 2-year disease-free survival without censoring was 78·9% (95% CI 63·2–88·4) versus 67·6% (51·5–79·4; appendix).

**Figure 4:**
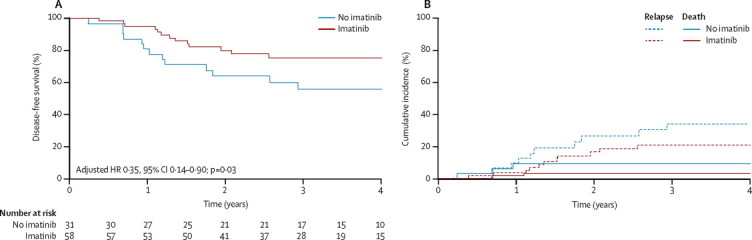
Disease-free survival curves and cumulative incidence of relapse and of death in continuous complete remission for good-risk patients, analysed as treated (A) Disease-free survival. (B) Cumulative incidence of relapse and death continuous complete remission for patients in the good-risk group. One event in a patient in the imatinib group at 6 years after randomisation is omitted (died in continuous complete remission of pulmonary graft-versus-host disease after transplantation).

The as-treated analyses compared 58 good-risk patients who received imatinib (including 12 assigned to the good-risk, no imatinib group), with 31 patients who did not. 4-year disease-free survival was 75·2% (95% CI 61·0–84·9) versus 55·9% (36·1–71·7), with an absolute difference of 19·3% (–2·0 to 41·0; p=0·06). HR adjusted by post-induction minimal residual disease was 0·35 (0·14–0·90; p=0·03; figure 4A). The cumulative incidence of relapse at 4 years was 21·2% (95% CI 9·8–32·6) for patients receiving imatinib and 34·4% (16·4–52·4) for those not receiving imatinib (p=0·21; figure 4B).

Of the 58 patients receiving imatinib, 11 did not have a transplant, of whom four relapsed. Of the 31 patients who did not receive imatinib, nine were not transplanted. Four of nine relapsed and one died in continuous complete remission. 13 of the 18 good-risk patients not included in the randomisation received imatinib, although not always according to protocol. None relapsed and three died in continuous complete remission (one after stem-cell transplantation in first complete remission).

**Figure 5:**
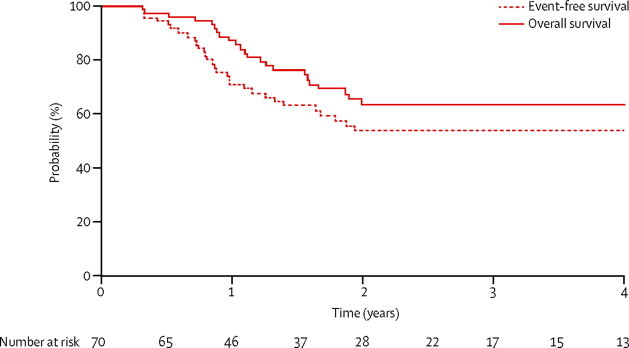
Survival in the poor-risk group An event that occurred after roughly 6 years is not shown (relapse in bone marrow and testis after transplantation in first complete remission).

Of the 70 poor-risk patients, 35 (50%) were not in complete remission at the end of induction. All patients eventually attained complete remission; 28 after protocol IB, five after HR1, and two after HR2. 59 (84%) received allogeneic stem-cell transplantation during first complete remission at a median time of 4·9 months (IQR 4·1–5·4). 4-year event-free survival was 53·5% (95% CI 40·4–65·0) and 4-year overall survival was 63·5% (95% CI 50·2–74·2; figure 5). 35 patients who were in complete remission at the end of induction had a 4-year event-free survival of 58·5% (95% CI 40·9–76·1) versus 48·9% (95% CI 31·5–49·1) for the remaining 35 who achieved complete remission at a later time (p=0·45). Disease-free survival at 4 years from complete remission was the same as event-free survival, because no patient was resistant to protocol.

23 patients relapsed (table 2) within 24 months of study entry, mostly involving bone marrow (n=21). Roughly 70% of these patients died because of disease progression. Eight patients died in continuous complete remission and none were judged to be related to imatinib.

Proportions of patients with the most commonly reported serious adverse events did not differ substantially between groups (p=0·64 when comparing good-risk patients treated with imatinib to those without) and were mainly related to myelosuppression (table 3). Infections were the most common serious adverse event. Minor delays (≤1 week) in administration of chemotherapy were more common in good-risk patients not receiving imatinib than those receiving imatinib (14/31 [45·2%] *vs* 13/58 [22·4%]) whereas major delays (>1 week) occurred in 25 of 58 (43·1%) patients in the good-risk, imatinib group versus six of 31 (19·4%) patients in the good-risk, no imatinib group. However, this difference was not significant (p=0·07). Poor-risk patients had similar results (20 had minor and 25 had major delays). 13 of 31 (41·9%) patients in the good-risk, no imatinib group, 26 of 58 (44·8%) patients in the good-risk, imatinib group, and 40 of 70 (57·1%) patients in the poor-risk group had a dose decrease of more than 10% for chemotherapy and imatinib cumulative doses between the beginning of protocol IB and the end of HR3.

**Table 3:** Serious adverse events

		**Good-risk, imatinib group (n=58)**	**Good-risk, no imatinib group (n=31)**	**Poor-risk group (n=70)**
Any primary system organ class		16 (28%)	10 (32%)	24 (34%)
Infections				
	Fungal infection	1 (2%)	1 (3%)	4 (6%)
	Localised infection	1 (2%)	2 (6%)	2 (3%)
	Other infection	5 (9%)	3 (10%)	9 (13%)
	Any*	7 (12%)	5 (16%)	14 (20%)
Vascular disorders				
	Deep vein thrombosis	0	0	0
Nervous system disorders				
	Cerebral thrombosis	0	0	0
	Convulsion	1 (2%)	1 (3%)	1 (1%)
	Paraesthesia	0 (0%)	0 (0%)	1 (1%)
	Cerebral haemorrhage	0 (0%)	0 (0%)	1 (1%)
	Any	1 (2%)	1 (3%)	3 (4%)
Gastrointestinal disorders				
	Pancreatitis	1 (2%)	0 (0%)	0 (0%)
	Gastrointestinal haemorrhage	0 (0%)	1 (3%)	0 (0%)
	Any	1 (2%)	1 (3%)	0 (0%)
Psychiatric disorders				
	Psychotic disorder	1 (2%)	0 (0%)	0 (0%)
Skin and subcutaneous tissue disorders				
	Dermatitis exfoliative	0	0	0
Metabolism and nutrition disorders				
	Diabetes mellitus	0	0	0
Immune system disorders				
	Anaphylactic shock	1 (2%)	1 (3%)	0 (0%)
Musculoskeletal and connective tissue disorders				
	Osteonecrosis	1 (2%)	1 (3%)	0 (0%)
Cardiac disorders				
	Cardiac failure	0 (0%)	0 (0%)	2 (3%)
	Arrhythmia	0	0	0
	Any	0	0	2 (3%)
Hepatobiliary disorders				
	Hepatic failure	1 (2%)	1 (3%)	1 (1%)
Renal and urinary disorders				
	Renal failure	0	0	0
	Renal impairment	1 (2%)	1 (3%)	0 (0%)
	Any	1 (2%)	1 (3%)	0 (0%)
Other		7 (12%)	3 (10%)	8 (11%)

A patient with more than one adverse events in a subcategory (eg, fungal infection) is counted only once under that subcategory. A patient with several adverse events during treatment is counted only once. The table includes data for all treatment phases and allogeneic stem-cell transplantation.

* Patients who had more than one subcategory of infection are only counted once in this row.

## Discussion

This study was designed to assess the efficacy and safety of imatinib when added to the Berlin–Frankfurt–Munster chemotherapy backbone in paediatric patients with Philadelphia-chromosome-positive ALL. Enrolment was stopped in 2009, because of the results of the COG study^21^ and unwillingness of clinicians and families to enrol after numerous publications on data for adults.^19^ Although the study is not powered to fully address the primary study aim, important conclusions can be derived.

All patients enrolled achieved complete remission, whereas previous reports of patients treated with the same chemotherapy backbone note that as many as 10% of patients were resistant to treatment.^3^ All poor-risk patients not in complete remission after induction had complete remission with imatinib during consolidation treatment. Disease-free survival was better in the good-risk, imatinib group than in the no imatinib group, despite the difference in minimal residual disease at baseline, which favoured patients not receiving imatinib. Only the as-treated analysis showed a better outcome for imatinib-treated patients after adjustment for differences in minimal residual disease (although with the caveat of potential selection bias). Additionally, toxic effects did not differ significantly between groups. Detailed, graded data for adverse events were not collected.

In a historical cohort of 286 patients, 4-year disease-free survival was 42·3% (95% CI 36·4–48·2); less than in our study (table 4). In the same cohort, 61% of patients were transplanted in first complete remission. Mortality related to transplantation diminished with time, accounting for 14% in the historical data and 9% in our study. In the historical cohort, poor-risk patients who had early response assessed by peripheral blood had a worse outcome than those who were assessed by bone marrow early response.^3^ This finding is supported by data from the historical cohort and our study, although the difference was not significant in our study (table 4).

**Table 4:** Comparison of outcome from EsPhALL with historical data

		**Historical data**^3^				**EsPhALL**				**p value for DFS**
		n	DFS (%; SE)	ASCT in first CR (n; %)	Treatment-related mortality from ASCT in first CR (n; %)	n	DFS (%; SE)	ASCT in first CR (n; %)	Treatment-related mortality from ASCT in first CR (n; %)	
Overall		286	42·3% (3·0%)	174 (61%)	25 (14%)	178	61·9% (4·1%)	137 (77%)	12 (9%)	0·0008
Poor risk		64	29·7% (6·3%)	43 (67%)	6 (14%)	70	53·5% (6·4%)	59 (84%)	6 (10%)	0·0092
Poor risk (early response in peripheral blood)		15	15 events (one at risk at 2 years)	7 (47%)	3 (43%)	39	43·6% (8·7%)*	32 (82%)	4 (13%)	..
Poor risk (early response in bone marrow)		49	38·8% (7·1%)*	36 (73%)	3 (8%)	27	60·8% (9·8%)*	23 (85%)	2 (9%)	..
Good risk		200	47·6% (3·6%)	115 (58%)	17 (15%)	90†	67·1% (5·3%)	69 (77%)	5 (7%)	0·0104
	No imatinib	..	..	..	..	44	61·7% (7·6%)	..	..	
Imatinib		..	..	..	..	46	72·9% (7·1%)	..	..	0·0141‡

Analyses of the historical cohort were done for the groups who later entered EsPhALL (AIEOP, BFM, COALL, DCOG, FRALLE, UK, NOPHO). This cohort includes 319 patients, of whom 31 (9·7%) were resistant and two (0·6%) died in induction, leaving 286 patients in first CR at the end of the protocol-specified induction period. 22 of 286 patients are not classified by risk because early response was unknown. DFS is at 4 years unless otherwise stated. DFS=disease-free survival. ASCT=allogeneic stem-cell transplantation. CR=complete remission.

* At 3 years.

† 18 good risk patients are excluded because they were not assigned.

‡ For historical, good risk versus good risk, imatinib.

Our results are concordant with those of the COG study^21^ and provide evidence of long-term benefit of treatment with imatinib for all patient groups (panel). However, EsPhALL has several differences from the COG AALL0031 study. COG reported that use of imatinib after induction negated the prognostic effect of end-of-induction minimal residual disease. In EsPhALL, early response to chemotherapy continued to be predictive—poor-risk patients still have an unfavourable outcome compared with good-risk patients. Patients with poor response to monotherapy with steroids as measured by blast cell count in peripheral blood, compared with bone marrow assessment early in induction, have a worse outcome (table 4), supporting historical findings.^3^ In view of the different induction protocols used in each study, interpretation of this variation is difficult. The prednisone poor-response group might be characteristic of the most highly resistant group of patients with Philadelphia-chromosome-positive ALL. Whether starting imatinib (or a second generation tyrosine-kinase inhibitor) during induction would cancel the negative effect of poor early response with steroids is still unclear.

Panel **Research in context**

**Systematic review**

We searched original English language publications in PubMed to May 16, 2012, with the terms “Ph+ALL”, “children”, and “TKI” or “imatinib”. We found two studies in which imatinib was assessed as part of first-line chemotherapy in children with acute lymphoblastic leukaemia. None were randomised. One was done by the Children's Oncology Study Group (COG)^21^ and the other by the Sociedad Española de Hematología y Oncología Pediátricas.^22^ In the COG study, an intensive chemotherapy regimen was given with imatinib (340 mg/m^2^ per day) administered with increasing exposure in five cohorts of patients, from 42 (cohort 1) to 280 continuous days (cohort 5) before maintenance. Patients with an HLA-identical sibling donor had allogeneic stem-cell transplantation in first complete remission and were then given imatinib for 6 months. 3-year event-free survival improved significantly for 44 patients in cohort 5 compared with historical controls, with no important toxic effects associated with imatinib. The outcome of transplanted patients was similar to patients in cohort 5 treated with chemotherapy plus imatinib. Sociedad Española de Hematología y Oncología Pediátricas has reported results of three consecutive trials since 1994.^22^ In the latest, (SHOP-2005) imatinib (260 mg/m^2^ per day) was given from day 15 of induction. Patients with an HLA-identical sibling or unrelated donor had allogeneic stem-cell transplantation in first complete remission (15 transplants in 16 imatinib patients *vs* 17 in 27 historical controls). 3-year event-free survival of patients who received imatinib was significantly higher than that of historical controls who received similar chemotherapy without imatinib.

**Interpretation**

The results of our study confirm the findings of previous observational studies, although imatinib resulted in an improvement only in the per-protocol analysis after adjustment for minimal residual disease and in patients with poor prognosis. Generally, imatinib was well tolerated with no increase in toxic effects in our study. The role of allogeneic stem-cell transplantation in first complete remission is unclear for patient with Philadelphia-chromosome-positive acute lymphoblastic leukaemia receiving imatinib, since most patients in our study had a transplant.

The central role of allogeneic stem-cell transplantation was established in a pre-imatinib large cohort of patients.^3^ However, COG reported^21^ that allogeneic stem-cell transplantation provides no benefit compared with treatment with intensive continuous imatinib (cohort 5). This finding, albeit from a small number of patients, holds true with longer follow-up (Schultz K, COG, personal communication). In EsPhALL, about 80% of enrolled patients had allogeneic stem-cell transplantation, with the limitation that a common policy of administration of tyrosine-kinase inhibitors after transplantation was not adopted. We do not know whether transplantation or tyrosine-kinase inhibitors explain the improvement compared with historical controls. In both good-risk and poor-risk groups, the few patients who received imatinib but not stem-cell transplantation, excluding those who failed before the median time to transplantation, had a poorer outcome (three of nine good-risk patients and five of seven poor-risk patients relapsed). However, this finding is limited by the small number of patients who did not have a transplant. That concomitant use of tyrosine-kinase inhibitors earlier, more continuously, or for longer, might negate the requirement for myeloablative therapy should be investigated further. In this context, serial analysis of minimal residual disease might help to select patients who can be treated with intensive chemotherapy protocols including a tyrosine-kinase inhibitor but without allogeneic stem-cell transplantation.

Our data support further investigation of new tyrosine-kinase inhibitors in conjunction with the established chemotherapy backbone for treatment of children with Philadelphia-chromosome-positive ALL. The EsPhALL network is assessing the effect of earlier, continuous, and longer exposure to imatinib and use of a second generation tyrosine-kinase inhibitor. Possible next steps are assessments of whether exposure to tyrosine-kinase inhibitors can change the intensity of chemotherapy and the use of transplantation in first remission.

## References

[1] Aricò M, Valsecchi MG, Camitta B (2000). Outcome of treatment in children with Philadelphia chromosome-positive acute lymphoblastic leukemia. N Engl J Med.

[2] Pui CH, Evans WE (2006). Treatment of acute lymphoblastic leukemia. N Engl J Med.

[3] Aricò M, Schrappe M, Hunger SP (2010). Clinical outcome of children with newly diagnosed Philadelphia chromosome-positive acute lymphoblastic leukemia treated between 1995 and 2005. J Clin Oncol.

[4] Schindler T, Bornmann W, Pellicena P, Miller WT, Clarkson B, Kuriyan J (2000). Structural mechanism for STI-571 inhibition of abelson tyrosine kinase. Science.

[5] Heinrich MC, Blanke CD, Druker BJ, Corless CL (2002). Inhibition of KIT tyrosine kinase activity: a novel molecular approach to the treatment of KIT–positive malignancies. J Clin Oncol.

[6] Druker BJ, Sawyers CL, Kantarjian H (2001). Activity of a specific inhibitor of the BCR-ABL tyrosine kinase in the blast crisis of chronic myeloid leukemia and acute lymphoblastic leukemia with the Philadelphia chromosome. N Engl J Med.

[7] Ottmann OG, Druker BJ, Sawyers CL (2002). A phase 2 study of imatinib in patients with relapsed or refractory Philadelphia chromosome-positive acute lymphoid leukemias. Blood.

[8] Thomas DA, Faderl S, Cortes J (2004). Treatment of Philadelphia chromosome-positive acute lymphocytic leukemia with hyper–CVAD and imatinib mesylate. Blood.

[9] Lee KH, Lee JH, Choi SJ (2005). Clinical effect of imatinib added to intensive combination chemotherapy for newly diagnosed Philadelphia chromosome-positive acute lymphoblastic leukemia. Leukemia.

[10] Yanada M, Takeuchi J, Sugiura I (2006). High complete remission rate and promising outcome by combination of imatinib and chemotherapy for newly diagnosed *BCR-ABL*-positive acute lymphoblastic leukemia: a phase II study by the Japan Adult Leukemia Study Group. J Clin Oncol.

[11] Wassmann B, Pfeifer H, Goekbuget N (2006). Alternating versus concurrent schedules of imatinib and chemotherapy as front-line therapy for Philadelphia-positive acute lymphoblastic leukemia (Ph+ ALL). Blood.

[12] Lee S, Kim YJ, Min CK (2005). The effect of first-line imatinib interim therapy on the outcome of allogeneic stem cell transplantation in adults with newly diagnosed Philadelphia chromosome-positive acute lymphoblastic leukemia. Blood.

[13] de Labarthe A, Rousselot P, Huguet-Rigal F (2007). Imatinib combined with induction or consolidation chemotherapy in patients with de novo Philadelphia chromosome-positive acute lymphoblastic leukemia: results of the GRAAPH–2003 study. Blood.

[14] Vignetti M, Fazi P, Cimino G (2007). Imatinib plus steroids induces complete remissions and prolonged survival in elderly Philadelphia chromosome-positive patients with acute lymphoblastic leukemia without additional chemotherapy: results of the Gruppo Italiano Malattie Ematologiche dell'Adulto (GIMEMA) LAL0201-B protocol. Blood.

[15] Ottmann OG, Wassmann B, Pfeifer H (2007). Imatinib compared with chemotherapy as front–line treatment of elderly patients with Philadelphia chromosome–positive acute lymphoblastic leukemia (Ph+ALL). Cancer.

[16] Ribera JM, Oriol A, Gonzalez M (2010). Concurrent intensive chemotherapy and imatinib before and after stem cell transplantation in newly diagnosed Philadelphia chromosome-positive acute lymphoblastic leukemia. Final results of the CSTIBES02 trial. Haematologica.

[17] Bassan R, Rossi G, Pogliani EM (2010). Chemotherapy-phased imatinib pulses improve long–term outcome of adult patients with Philadelphia chromosome–positive acute lymphoblastic leukemia: Northern Italy Leukemia Group protocol 09/00. J Clin Oncol.

[18] Mizuta S, Matsuo K, Yagasaki F (2011). Pre-transplant imatinib-based therapy improves the outcome of allogeneic hematopoietic stem cell transplantation for BCR-ABL-positive acute lymphoblastic leukemia. Leukemia.

[19] Fielding AK, Buck G, Lazarus H (2010). Imatinib significantly enhances long-term outcomes in Philadelphia positive acute lymphoblastic leukemia; final results of the UKALLXII/ECOG2993 Trial. Blood.

[20] Pfeifer H, Goekbuget N, Volp C (2010). Long–term outcome of 335 adult patients receiving different schedules of imatinib and chemotherapy as front–line treatment for Philadelphia-positive acute lymphoblastic leukemia (Ph+ALL). Blood.

[21] Schultz KR, Bowman WP, Aledo A (2009). Improved early event-free survival with imatinib in Philadelphia chromosome-positive acute lymphoblastic leukemia: a Children's Oncology Group Study. J Clin Oncol.

[22] Rives S, Estella J, Gómez P (2011). Intermediate dose of imatinib in combination with chemotherapy followed by allogeneic stem cell transplantation improves early outcome in paediatric Philadelphia chromosome-positive acute lymphoblastic leukaemia (ALL): results of the Spanish Cooperative Group SHOP studies ALL-94, ALL-99 and ALL-2005. Br J Haematol.

[23] Campana D (2012). Minimal residual disease monitoring in childhood acute lymphoblastic leukemia. Curr Opin Hematol.

[24] Schrappe M, Aricò M, Harbott J (1998). Philadelphia chromosome-positive (Ph+) childhood acute lymphoblastic leukemia: good initial steroid response allows early prediction of a favorable treatment outcome. Blood.

[25] Valsecchi MG, Silvestri D, Covezzoli A, De Lorenzo P (2008). Web-based international studies in limited populations of pediatric leukemia. Ped Blood Cancer.

[26] Marks DI, Bird JM, Cornish JM (1998). Unrelated donor bone marrow transplantation for children and adolescents with Philadelphia-positive acute lymphoblastic leukemia. J Clin Oncol.

[27] Champagne MA, Capdeville R, Krailo M (2004). Imatinib mesylate (STI571) for treatment of children with Philadelphia chromosome-positive leukemia: results from a Children's Oncology Group phase 1 study. Blood.

[28] Flohr T, Schrauder A, Cazzaniga G (2008). Minimal residual disease-directed risk stratification using real-time quantitative PCR analysis of immunoglobulin and T-cell receptor gene rearrangements in the international multicenter trial AIEOP-BFM ALL 2000 for childhood acute lymphoblastic leukemia. Leukemia.

[29] Gabert J, Beillard E, van der Velden VH (2003). Standardization and quality control studies of ‘real–time’ quantitative reverse transcriptase polymerase chain reaction of fusion gene transcripts for residual disease detection in leukemia—a Europe Against Cancer program. Leukemia.

[30] Conter V, Bartram CR, Valsecchi MG (2010). Molecular response to treatment redefines all prognostic factors in children and adolescents with B-cell precursor acute lymphoblastic leukemia: results in 3184 patients of the AIEOP-BFM ALL 2000 study. Blood.

[31] Schrappe M, Valsecchi MG, Bartram CR (2011). MRD response determines relapse risk overall and in subsets of childhood T-cell ALL: results of the AIEOP-BFM_ALL 2000 study. Blood.

[32] Lachin JM, Faulkes MA (1986). Evaluation of sample size and power for analyses of survival with allowance of nonuniform patient entry, losses to follow-up, non compliance and stratification. Biometrics.

[33] Greenwood M (1926). A report on the natural duration of cancer. Reports on public health and medical subjects.

