# Supplementary material for: Imatinib after induction for treatment of children and adolescents with Philadelphia-chromosome-positive acute lymphoblastic leukaemia (EsPhALL): a randomised, open-label, intergroup study
Source: Lancet Oncol. 2012 Sep;13(9):936–45. doi: 10.1016/S1470-2045(12)70377-7 (PMC3431502; doi:10.1016/S1470-2045(12)70377-7)
Supplement: Supplementary appendix [file mmc1.pdf]

THE LANCET Oncology

Supplementary appendix

This appendix formed part of the original submission and has been peer reviewed. We post it as supplied by the authors.

Supplement to: Biondi A, Schrappe M, De Lorenzo P, et al. Imatinib after induction for treatment of children and adolescents with Philadelphia-chromosome-positive acute lymphoblastic leukaemia (EsPhALL): a randomised, open-label, intergroup study. *Lancet Oncol* 2012; published online Aug 14. http://dx.doi.org/10.1016/S1470-2045(12)70377-7.

Supplementary Table 1. Recruitment overall, by risk group and assigned arm (in Good Risk) and by participating group

Participating Group	Recruitment		Good Risk Randomized study		Assigned arm GR-noIM	Assigned arm GR-IM	Poor Risk Observational study	
	N	%	N	%	N	N	N	%
**AIEOP**	27	15·17	13	14·44	6	7	12	17·14
**BFM-G***	41	23·0	20	22·2	9	11	14	20·00
**COALL^**	7	3·93	4	4·44	2	2	3	4·29
**CPH**	9	5·06	2	2·22	1	1	5	7·14
**DCOG**	12	6·74	8	8·89	4	4	3	4·29
**FRALLE^**	14	7·87	7	7·78	3	4	3	4·29
**MRC^**	38	21·35	21	23·33	11	10	17	24·29
**NOPHO^**	21	11·80	12	13·33	6	6	7	10·00
**Hong-Kong**	3	1·69	1	1·11	1	0	2	2·86
**PINDA**	6	3·37	2	2·22	1	1	4	5·71
**Total**	178		90		44	46	70	

GR-noIM= Good Risk patients randomized not to receive imatinib. GR-IM= Good Risk patients randomized to receive imatinib. N=number of patients. *includes 1 patients enrolled by BFM-Austria. ^these groups assessed early response in the bone marrow, while the remaining evaluated the peripheral blood.

Supplementary Table 2. Treatment phases of the EsPhALL protocol

Treatment phase	Drug	Single dose	Administration		Total dose per phase
			Day	Route	
**Protocol IB**	Cyclophosphamide<br>6-Mercaptopurine<br>Cytosine Arabinoside<br><br>Methotrexate<br><br><br>Imatinib*	1000 mg/m^2<br>60 mg/m^2<br>75 mg/m^2<br><br>≥1 year < 2 year = 8 mg<br>≥2 years <3 years = 10 mg<br>≥3 years = 12 mg<br>300 mg/m^2	1, 28<br>1-28<br>3-6, 10-13, 17-20, 24-27<br>3, 7<br><br><br>1-28	iv<br>oral<br>sc<br><br>it<br><br><br>oral	2000 mg/m^2<br>1680 mg/m^2<br>1200 mg/m^2<br><br>age-related<br><br><br>8400 mg/m^2
**Consolidation block HR1**	Dexamethasone<br>Vincristine<br>Methotrexate<br>Cytosine Arabinoside<br>L-Asparaginase<br>Cyclophosphamide<br>Methotrexate (MTX)<br>Cytosine Arabinoside (ARA-C)<br>Prednisone (PRED)<br><br>Imatinib*	20 mg/m^2<br>1·5 mg/m^2<br>5000 mg/m^2<br>2000 mg/m^2<br>25000 IU/m^2<br>200 mg/m^2<br>≥1 year < 2 year = MTX 8 mg ARA-C 20 mg PRED 6 mg<br>≥2 years <3 years = MTX 10 mg ARA-C 26 mg PRED 8 mg<br>≥3 years = MTX 12 mg ARA-C 30 mg PRED 10 mg<br>300 mg/m^2	1-5<br>1,6<br>1<br>5<br>6<br>2-4<br>1<br><br><br><br>6-20	oral or iv<br>iv<br>iv<br>iv<br>im<br>iv<br>it<br><br><br><br>oral	100 mg/m^2<br>3 mg/m^2<br>5000 mg/m^2<br>4000 mg/m^2<br>25000 IU/m^2<br>1000 mg/m^2<br>age-related<br><br><br><br>4200 mg/m^2
**Consolidation block HR2**	Dexamethasone<br>Vindesine<br>Methotrexate<br>Ifosphamide<br>L-Asparaginase<br>Daunorubicin<br>Methotrexate (MTX)<br>Cytosine Arabinoside (ARA-C)<br>Prednisone (PRED)<br><br>Imatinib*	20 mg/m^2<br>3 mg/m^2<br>5000 mg/m^2<br>800 mg/m^2<br>25000 IU/m^2<br>30 mg/m^2<br>≥1 year < 2 year = MTX 8 mg ARA-C 20 mg PRED 6 mg<br>≥2 years <3 years = MTX 10 mg ARA-C 26 mg PRED 8 mg<br>≥3 years = MTX 12 mg ARA-C 30 mg PRED 10 mg<br>300 mg/m^2	1-5<br>1,6<br>1<br>2-4<br>6<br>5<br>1<br><br><br><br>6-20	oral or iv<br>iv<br>iv<br>iv<br>im<br>iv<br>it<br><br><br><br>oral	100 mg/m^2<br>6 mg/m^2<br>5000 mg/m^2<br>4000 mg/m^2<br>25000 IU/m^2<br>30 mg/m^2<br>age-related<br><br><br><br>4200 mg/m^2
**Consolidation block HR3**	Dexamethasone<br>Cytosine Arabinoside<br>Vepeside<br>L-Asparaginase<br>Methotrexate (MTX)<br>Cytosine Arabinoside (ARA-C)<br>Prednisone (PRED)<br><br>Imatinib*	20 mg/m^2<br>2000 mg/m^2<br>100 mg/m^2<br>25000 IU/m^2<br>≥1 year < 2 year = MTX 8 mg ARA-C 20 mg PRED 6 mg<br>≥2 years <3 years = MTX 10 mg ARA-C 26 mg PRED 8 mg<br>≥3 years = MTX 12 mg ARA-C 30 mg PRED 10 mg<br>300 mg/m^2	1-5<br>1-2<br>3-5<br>6<br>1<br><br><br><br>6-20	oral or iv<br>iv<br>iv<br>im<br>it<br><br><br><br>oral	100 mg/m^2<br>4000 mg/m^2<br>500 mg/m^2<br>25000 IU/m^2<br>age-related<br><br><br><br>4200 mg/m^2
**Reinduction Protocol II****	Dexamethasone<br>Vincristine<br>Doxorubicin<br>L-Asparaginase<br>Cyclophosphamide<br>6-Thioguanine<br>Cytosine Arabinoside<br>Methotrexate<br><br><br>Imatinib*	10 mg/m^2<br>1·5 mg/m^2<br>25 mg/m^2<br>10000 IU/m^2<br>1000 mg/m^2<br>60 mg/m^2<br>75 mg/m^2<br>≥1 year < 2 years = 8 mg<br>≥2 years <3 years = 10 mg<br>≥3 years = 12 mg<br>300 mg/m^2	1-21 + tapering<br>8,15,22,29<br>8,15,22,29<br>8,11,15,18<br>36<br>36-49<br>38-41, 45-48<br>38, 45<br><br><br>36-63	oral<br>iv<br>iv<br>im<br>iv<br>oral<br>sc<br>it<br><br><br>oral	235 mg/m^2<br>6 mg/m^2<br>100 mg/m^2<br>40000 IU/m^2<br>1000 mg/m^2<br>840 mg/m^2<br>600 mg/m^2<br>age-related<br><br><br>8400 mg/m^2
**Interim Maintenance**	6-Mercaptopurine<br>Methotrexate<br>Cranial irradiation	50 mg/m^2<br>20 mg/m^2<br>1·4-1·7 Gy	1-29<br>8,15,22,29	oral<br>oral	1450 mg/m^2<br>80 mg/m^2<br>Standard: 18 Gy<br>- if < 2 years: 12 Gy<br>- if CNS inv.: 24 Gy
**Continuation therapy Maintenance**	6-Mercaptopurine<br><br><br>Methotrexate	50 mg/m^2<br><br><br>20 mg/m^2	Daily till day +728 from diagnosis<br>weekly till day +728 from diagnosis	oral<br><br><br>oral	

iv=intravenous; im=intramuscolar; it=intrathecal; sc=subcutaneous. [*]imatinib was administered to all Poor Risk patients and to Good Risk patients in a randomized fashion. [**] Reinduction Protocol II was administered twice, one before and one after Interim Maintenance. During the second administration, intrathecal therapy was omitted due to previous cranial irradiation.

Supplementary Figure 1. Disease-free survival curves with 2-year estimates (standard error) by randomized arm in Good Risk(ITT analysis), censoring at allo-SCT in CR1

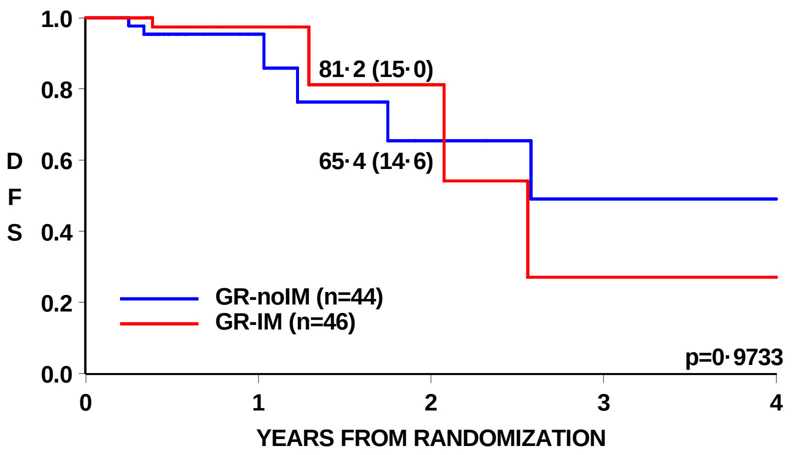

Number at risk

GR-noIM	44	19	10	8	5	4	2	2	2
GR-IM	46	16	7	5	4	2	2	2	2

ITT=intention to treat. allo-SCT= allogeneic stem cell transplantation. CR1=First complete remission. GR-noIM= Good Risk patients randomized not to receive imatinib. GR-IM= Good Risk patients randomized to receive imatinib.
